# ‘Your heart is resting and pumping at the same time’: Mental health impact of seeking asylum among sexual minority men

**DOI:** 10.1177/14034948241251553

**Published:** 2024-05-22

**Authors:** Maria Gottvall, Rummage Isaac, Ronah Ainembabazi, Sumera Yasin, Anna Eldebo, Tommy Carlsson

**Affiliations:** 1 The Department of Women’s and Children’s Health, Uppsala University, Sweden and; 2 The Department of Health Sciences, The Swedish Red Cross University, Sweden

**Keywords:** Asylum seekers, cisgender men, forced migrants, mental health, sexual and gender minorities

## Abstract

**Aims:**

To explore the experiences of seeking asylum and its impact on mental health among sexual minority forced migrant cisgender men living in Sweden.

**Methods:**

Exploratory qualitative study based on individual semi-structured interviews with 15 adult gay and bisexual cisgender men recruited via a combination of purposeful, convenience and snowball sampling. Data were analysed with systematic text condensation through a collaborative approach with three migrants with lived experience.

**Results:**

Seeking asylum had been an emotionally challenging journey for the participants in this study, involving several procedures that negatively impacted mental health. Being expected to disclose intimate information during asylum interviews had been a significant challenge, alongside needing to wait through long periods in uncertainty with little information about the progress of their asylum case. The behaviours and attitudes of professionals involved in the legal procedures had been a central aspect, as participants encountered interpreters and caseworkers who acted disrespectful and homophobic during asylum interviews. Participants mentioned that the behaviours of interpreters and the accuracy of the interpretation could influence the outcome of asylum claims and how comfortable they felt in sharing information. Thus, participants emphasized the importance of adequate and accurate interpreter services.

**Conclusions:**

**Sexual minority men are faced with an unfamiliar and emotionally challenging position when seeking asylum and undergoing asylum interviews. The findings highlight the importance of adequate competence among professionals involved in asylum interviews, including interpreter utilization. Research is needed to determine effective methods to support these men throughout their asylum process.**

## Background

Globally, individuals of sexual minorities face significant danger and violence [1], leaving many with no other alternative than to flee and seek asylum [2]. Seeking asylum requires forced migrants to undergo several interviews to confirm the credibility of seeking protection [3]. Studies call attention to the risk of psychological distress among sexual minority forced migrants during the asylum process [3,4], including the mental health consequences of being asked to describe and relive traumatic experiences [5,6]. Consequently, it is essential that forced migrants are given adequate support and opportunities to present their case during asylum interviews [7]. For sexual minority forced migrants, an added layer of shame and internalized homophobia may hinder them from speaking openly about their experiences and identities [8].

Creating a reassuring and safe setting during asylum interviews is essential and emphasized in international guidelines [9]. However, reports suggest that sexual minority migrants encounter disbelief among caseworkers and need to act according to stereotypical assumptions [10–12]. While the Nordic countries recognize persecution because of sexual orientation as a valid reason for asylum, empirical studies based on the Nordic context are scarce [10,11]. More than 12,000 migrants sought asylum in Sweden during 2022. The number of sexual minority asylum seekers and undocumented migrants is unknown. Asylum seekers are interviewed by caseworkers, who determine the need for public counsels and interpreters. Understanding the unique mental health challenges faced by sexual minority forced migrants in Sweden is crucial for informing public health interventions that foster mental health of asylum seekers and that promote an equitable asylum process.

The aim of this study was to explore the experiences of seeking asylum and its impact on mental health among sexual minority forced migrant cisgender men living in Sweden. Sexual orientation can be viewed as an enduring physical, romantic, emotional and/or spiritual attraction to another person. Hereafter, the term sexual minorities is used to refer to people whose sexual orientation is not self-identified as exclusively heterosexual.

## Methods

### Study design

This was an exploratory qualitative study with an inductive approach. The study is part of a public contribution research project based on collaboration between two researchers and three sexual minority forced migrants with lived experience, henceforth referred to as experts by lived experience [13]. This study was approved by the Swedish Ethics review authority (approval number: 2022-01483-01). All participants provided written informed consent and received a gift card of SEK500.

### Participants

Adult forced migrants self-identifying as sexual minority cisgender men were recruited through a combination of convenience (online advertisements written in English, distributed via the social media and website of the university and research project), purposeful (through the networks of the research team) and snowball sampling. Persons interested in participating were referred to a digital application form; alternatively they were asked to call a research assistant. Potential participants were contacted to schedule a time and place for the interview, depending on their preferences. Recruitment took place between April and June 2023. The final sample consisted of 15 participants originating from Uganda (*n*=6), Ethiopia (*n*=2), Pakistan (*n*=2), Bangladesh, Eritrea, Iran and Rwanda (one not disclosing country of origin). Time in Sweden ranged from two months to five years (median: 11 months). The age of participants ranged between 20 and 47 years (median: 28). Highest level of education included university/college (*n*=8), high school (*n*=6) and elementary school (*n*=1). Fourteen identified as gay and one as bisexual.

### Data collection

Individual interviews were carried out by the first and last authors (registered nurse-midwives and researchers). Based on the preferences of the participants, the interviews were conducted in English and no participant desired an interpreter. A semi-structured topic guide was used, including the main question, ‘Please tell me about how your situation has been since you came to Sweden’ and follow-up questions (e.g. ‘How has your mental health been?’, ‘What challenges have you experienced?’). Participants were asked to describe their trajectory after arriving in Sweden, including their experiences of seeking asylum. At the end of the interview, the interviewer summarized the information to address misunderstandings. Seven interviews were carried out at the university campus, seven at a hotel and one via a digital video conference tool. The interviews lasted between 34 and 127 min (median: 64). Interviews were audio-recorded and transcribed verbatim.

### Data analysis

A collaborative analytic approach was applied, involving a team of two researchers and three experts by lived experience. The material was analysed with systematic text condensation, a stepwise procedure to analyse qualitative data [14]. The analysis involved four iterative steps (Step 1: preliminary themes identified; Step 2: meaning units identified and sorted into code groups; Step 3: meaning units sorted into subgroups, condensates written for each subgroup, illustrative quotes identified; Step 4: synthesized statements written and category headings produced). The team had continual discussions throughout each step until analysts agreed that the findings accurately reflected the content within the transcripts. Analysts represent a range of backgrounds and identities, including persons with diverse sexual orientations, gender identities and countries of origin. Two clinical psychologists were involved as consultants in the first and the last step of the analysis.

## Results

Seeking asylum had been an emotionally challenging journey with an internal turbulence related to disclosure of past experiences and waiting in uncertainty. Undergoing asylum interviews involved a sensitive dance of support and strain, as behaviours and attitudes of professionals had been a central component of interviews. While positive experiences of interaction with professionals had promoted comfortability and disclosure, some participants encountered disrespectful and homophobic behaviours when trying to present their case. Seven were waiting for the asylum decision, five had been granted, two had been denied and had appealed, and one had been denied after an appeal.

### Category 1: Emotional turbulence of disclosure and waiting

#### Caught in limbo while living with the agony of waiting

In common with other asylum seekers, participants experienced a challenging journey when needing to wait long periods in uncertainty. Complex international regulations (e.g. the Dublin regulation) resulted in unsafe periods living as an undocumented migrant. When asylum was sought, some needed to wait long periods before being called for interviews. After the final interview, participants waited several months in emotional turmoil before receiving a decision. Waiting for progress resulted in frustration and a reduced sense of hope. Eventually, some participants started questioning whether something was wrong with them or their case.

My lawyer told me like, it will take maybe 28 months for the court to reply. I can’t wait 28 months in the camp. So, that’s why I returned back to [city], just to find a job, you know. And to wait for the court. Imagine waiting for 28 months. It’s crazy, to be honest. (Participant 1)

Long waiting with little information about the progress induced stress, panic attacks, anxiety and sleeping difficulties. The waiting was referred to as ‘more than frustrating, it is the worst’, illustrating the mental health impact when left waiting in uncertainty. Recurrent worries and fears about what would happen if denied asylum had been a major concern. Having to wait in uncertainty had been so stressful for some that they considered leaving the host country and risking their life elsewhere. Some expressed that they would rather be killed or kill themselves than return to their country of origin.

There are times that I get depressed. You think a lot about waiting for your decision. You think a lot. You wonder if they reject you, what are you going to do. [Interviewer: Do you worry?] Yeah, sure. Of course. I hope for the best, but then that, on the other hand, you have to think of that too. Just in case if this happens, what will I do? How am I going to survive? (Participant 2)

#### An emotional whirlwind of revealing past experiences and current expectations

Participants experienced a significant emotional toll when undergoing repeated and lengthy asylum interviews, highlighting the difficult and unrealistic task of explaining thoughts, feelings and sexuality. Before asylum interviews, participants felt nervous and worried about questions they would be asked. Being asked highly detailed questions about their sexual orientation and traumatic experiences had been a significant and unfamiliar challenge. This had been in stark contrast to how they previously needed to conceal part of their identity while in their country of origin.

Some things you never thought you would say. They end up asking you about it, like parents, and it’s a sad feeling, so you don’t need to. . . you didn’t want to go there. It doesn’t feel good. [. . .] A lot of things go on in your mind and you just go around thinking about it. You just feel your heart resting and pumping at the same time. (Participant 3)

During asylum interviews, participants were expected to talk freely and openly about very difficult experiences and topics. Asylum interviews felt increasingly inquisitive, referred to as going ‘deeper and deeper’. Questions had been emotionally demanding when reminded of the significant traumas faced in the country of origin. For some participants, this exacerbated mental health burdens, such as post-traumatic stress and sleeping difficulties.

This second interview, it was okay but very hard. It was getting me this PTSD and [I was] scared more, more, more. I couldn’t sleep. [. . .] The last interview that I had, it was way worse, because everything [was] getting out. (Participant 4)

Once the final decision came, migrants who were granted asylum were overjoyed, feeling that it came as a surprise. When receiving the positive decision, they cried in happiness and disbelief. The decision made all the challenges worthwhile, with participants now being eager to continue as law-abiding citizens.

Within three or four months, I got my residency. I was the luckiest person at that time. No one ever believe that it’s even going to happen. I wouldn’t say that I had a worse experience or a bad experience. There were some things which happened after, but in the beginning, it was really like a fairytale. (Participant 5)

### Category 2: A sensitive dance with professionals during asylum interviews

#### The importance of affirming and accepting support in a setting you feel safe in

The importance of comfort and support during asylum interviews was highlighted by participants. They called attention to the vulnerable and exposed situation experienced when expected to share intimate and detailed information. Participants described varied quality of the communication with professionals involved. At the first interview, trying to speak openly about their persecution experiences and identity required significant confidence. Several received valuable support from their lawyer, who helped prepare for interviews, uplifted their spirits and made sure they were listened to. They helped participants review protocols and informed them about decisions. Some had been assigned a lawyer associated with organizations supporting sexual minority migrants, which meant they had specific knowledge and competence.

[Lawyer] prepares you very, very well before the interview. He calls you to his office and you have to go through you know, you have to narrate your story. [. . .] To tell your story is difficult, it’s complicated, so you have to be guided to follow the right procedures. (Participant 6)

The importance of being interviewed in a setting in which they felt safe was emphasized. The possibility of being open and honest during asylum interviews was influenced by the background and characteristics of the professionals conducting the interviews. Encountering caseworkers and interpreters originating from the same context or country as themselves had been challenging, as participants felt less safe and hindered to disclose intimate information. Moreover, some expressed that caseworkers acted in a way that signalled that their accounts were insufficient and not truthful. Presenting their case was sometimes further complicated by language barriers.

I didn’t expect my case officer was from my country [. . .] After that, I didn’t want to say something about my case [. . .] Because she’s from my country maybe if she will find I am gay, she tells other persons and I think she will say negative [things]. (Participant 7)

#### Dismissive and judgmental attitudes among professionals can hinder disclosure

Varied behaviours and attitudes among professionals involved in the asylum interviews were described, as some felt respected while others experienced homophobic and disrespectful attitudes. Participants expressed that it felt unlikely to get both a lawyer and an interpreter who are competent in interviewing sexual minority migrants. Some caseworkers acted dismissively and interrupted participants during the asylum interview, which made it difficult to give a full account of their asylum claim.

Sometimes they ask you a question in the middle of it, that it stops you from. . . or you forget the part you were [saying] [. . .] And you don’t say everything. And it’s a bad thing, because you need. . . If they ask you to say what really went on, they have to give you the time. (Participant 3)

The importance of sufficient competence and respectfulness of interpreters was emphasized, as their behaviours was considered to heavily influence the quality of asylum interviews. While some had positive experiences, others expressed that the behaviours of interpreters made them feel uncomfortable and unable to share information. Some interpreters had acted surprised, shocked and appeared judgmental when sexual orientation was mentioned. Being assigned an interpreter who had insufficient competence in sexual minorities and who showed disrespect led to participants’ leaving out important information.

Migration Agency brought me one translator. And you can feel, you know, from the way how. . . when I started talk about my sexuality, they were like, they were judging me [. . .] I was comfortable talking about my sexuality, but he was not comfortable to translate it. [. . .] When he started to translate, it was like, it didn’t make any sense. Even my lawyer after three hours, she said ‘No, we are not going to do this’. (Participant 1)

After asylum interviews, some participants realized that the protocol contained significant misinterpretations. This made them worried about their future, and they started to question the accuracy of the information that the decision would be based on. To make sure that their stories were heard authentically and truthfully, some decided to speak English during asylum interviews despite not being well-versed in the language.

[Caseworker] was asking me about my inside things, like, I need to express more about my inside. So, when I expressed, that translator was showing me, like, he’s so surprised [. . .] When my lawyer gave [me] protocol also, he changed almost everything. And I tell her, I don’t explain like that, and she helped by fixing these things. [Interviewer: So, he twisted your words?] I think, yes. (Participant 7)

## Discussion

This study contributes novel in-depth findings grounded in the Nordic context. Previous research reports that asylum interviews can aggravate psychological distress when participants are required to relive past traumas [6,15]. Many sexual minority forced migrants have faced significant pre-migration oppression and violence [1]. Retraumatization by having to tell their story over and over in an unsafe or non-affirming setting can lead to an increased risk for suicidality [16]. The findings highlight the vulnerability and emotional challenges within the target population when expected to reveal intimate information and talk openly.

Mental health of asylum seekers is impacted by a disruption of context and time, without a clear direction and waiting in passivity [17]. Our study further confirms previous research that sexual minority forced migrants feel caught in a no-man’s-land of waiting and fear potential consequences of deportation [10,18]. Having to wait a long time for a decision on an asylum claim can negatively impact mental health [19,20]. Sexual minorities face considerable danger to themselves and their family members if they were to be deported [1]. The risk of deportation can result in migrants having no choice than to live undocumented, which can lead to further exploitation and health burdens [21]. There is a need for more research to investigate methods to support sexual minority forced migrants when they experience uncertainty during waiting.

Establishing a reassuring and safe setting during asylum interviews is emphasized in international guidelines [9]. However, participants described instances of encountering dismissive and disrespectful behaviours among professionals, which had been emotionally difficult and resulted in barriers to presenting a full account of their claim. In line with our findings, studies highlight that sexual minority asylum seekers can be met with disbelief among caseworkers and may need to act according to stereotypical assumptions [10–12]. In striving to convince authorities some migrants can be driven to desperate measures, including going public about their sexuality and producing evidence of sexual acts [10]. Accurate interpretation is fundamental for asylum decisions. While limited studies have reported similar concerns about interpreter utilization [10,22], more research is needed. Our findings highlight the specific considerations needed when interviewing sexual minority asylum seekers. Taken together, the findings highlight the importance of respectful and non-judgmental support through a culturally sensitive approach. Our findings emphasize the importance of clear information about interpreter confidentiality.

We utilized diverse recruitment strategies to ensure a varied sample. While we believe we successfully achieved this in many regards, it is worth noting that participants resided in densely populated areas in central Sweden and most identified as gay men. Additional research based on more diverse samples would complement this study. Two Swedish-born researchers conducted the interviews, and it is possible that interviews would have generated richer data if peer interviews had been conducted [23]. No participant wanted an interpreter and it is possible that language barriers impacted interviews. Qualitative analyses are shaped by the preconceptions of the analysts. To approach the data from additional perspectives, the researchers engaged in co-analysis with experts by lived experience. Our intention was to reach enriched and nuanced results [24], and we argue that the collaborative approach strengthens the trustworthiness of the findings.

## Conclusions

When seeking asylum, sexual minority forced migrant men experience mental health burdens related to having their identity scrutinized, reliving traumatic experiences and having to wait for long periods in uncertainty. Going through intense and detailed asylum interviews requires a respectful and supportive interview setting. When homophobic attitudes are encountered, asylum seekers feel hindered in sharing valuable information and experience psychological distress. To promote comfort and accuracy, careful considerations are needed regarding interpreter utilization. More knowledge is needed about how to effectively provide psychosocial support for these men as they wait for progress in their asylum case.
